# Seafood from a changing Arctic

**DOI:** 10.1007/s13280-017-0954-2

**Published:** 2017-10-27

**Authors:** Max Troell, Arne Eide, John Isaksen, Øystein Hermansen, Anne-Sophie Crépin

**Affiliations:** 1 The Beijer Institute of Ecological Economics, The Royal Swedish Academy of Sciences, Lilla Frescativägen 4, 104 05 Stockholm, Sweden; 2 The Stockholm Resilience Centre, Stockholm University, Kräftriket 2 B, 10691 Stockholm, Sweden; 3 Norwegian College of Fishery Science , UiT – The Arctic University of Norway, Breivika, 9037 Tromsø, Norway; 4 Industrial Economics, Nofima, P.O. box 6122, 9291 Tromsø, Norway

**Keywords:** Arctic marine food web, Aquaculture, Capture fisheries, Climate change

## Abstract

We review current knowledge about climate change impacts on Arctic seafood production. Large-scale changes in the Arctic marine food web can be expected for the next 40–100 years. Possible future trajectories under climate change for Arctic capture fisheries anticipate the movement of aquatic species into new waters and changed the dynamics of existing species. Negative consequences are expected for some fish stocks but others like the Barents Sea cod (*Gadus morhua*) may instead increase. Arctic aquaculture that constitutes about 2% of global farming is mainly made up of Norwegian salmon (*Salmo salar*) farming. The sector will face many challenges in a warmer future and some of these are already a reality impacting negatively on salmon growth. Other more indirect effects from climate change are more uncertain with respect to impacts on the economic conditions of Arctic aquaculture.

## Introduction

Future climate development predictions indicate that we can expect large-scale changes in the Arctic marine food web the next 40–100 years (Hoegh-Guldberg et al. 2014). Possible future trajectories under climate change for capture fisheries (Eide 2016) and for Arctic aquaculture (Hermansen and Troell 2012) anticipate the movement of aquatic species into new waters, changes in dynamics of existing species, changes in management regimes and new regulations for novel commercial fisheries and aquaculture. Negative consequences are expected for traditional hunting of marine mammals, but for fish populations like Atlantic cod (*Gadus morhua*) in the Barents Sea environmental carrying capacity may instead increase (Perry et al. 2005; Eide 2017). Arctic aquaculture is an economically important business and makes up more than 50% of European Union volumes. The vast majority of this is Atlantic salmon (*Salmo salar*) farmed in Norway. The sector may face many challenges in a warmer future and some of these, being directly related to temperature increase, are already a reality for the industry. Other more indirect impacts will have more uncertain influence on the economic conditions of Arctic aquaculture production (Hermansen and Troell 2012).

Climate change affects food production and food security in the Arctic in complex ways. It encompasses many different dimensions, including health, pollution and globalisation through integrated markets. To date, there exists no pan-Arctic assessment that provides an overall picture (Arctic Council 2013). Box 1 presents a snapshot of the broader picture, but this paper limits its aims to reviewing and discussing possible impacts from climate change on Arctic industrial capture fisheries and aquaculture production. These are some of the most important industries in the Arctic and together they constitute relatively large shares of the gross domestic product (GDP) in some countries (e.g. 15% in Greenland and 10% in Iceland).

**Fig. 1:**
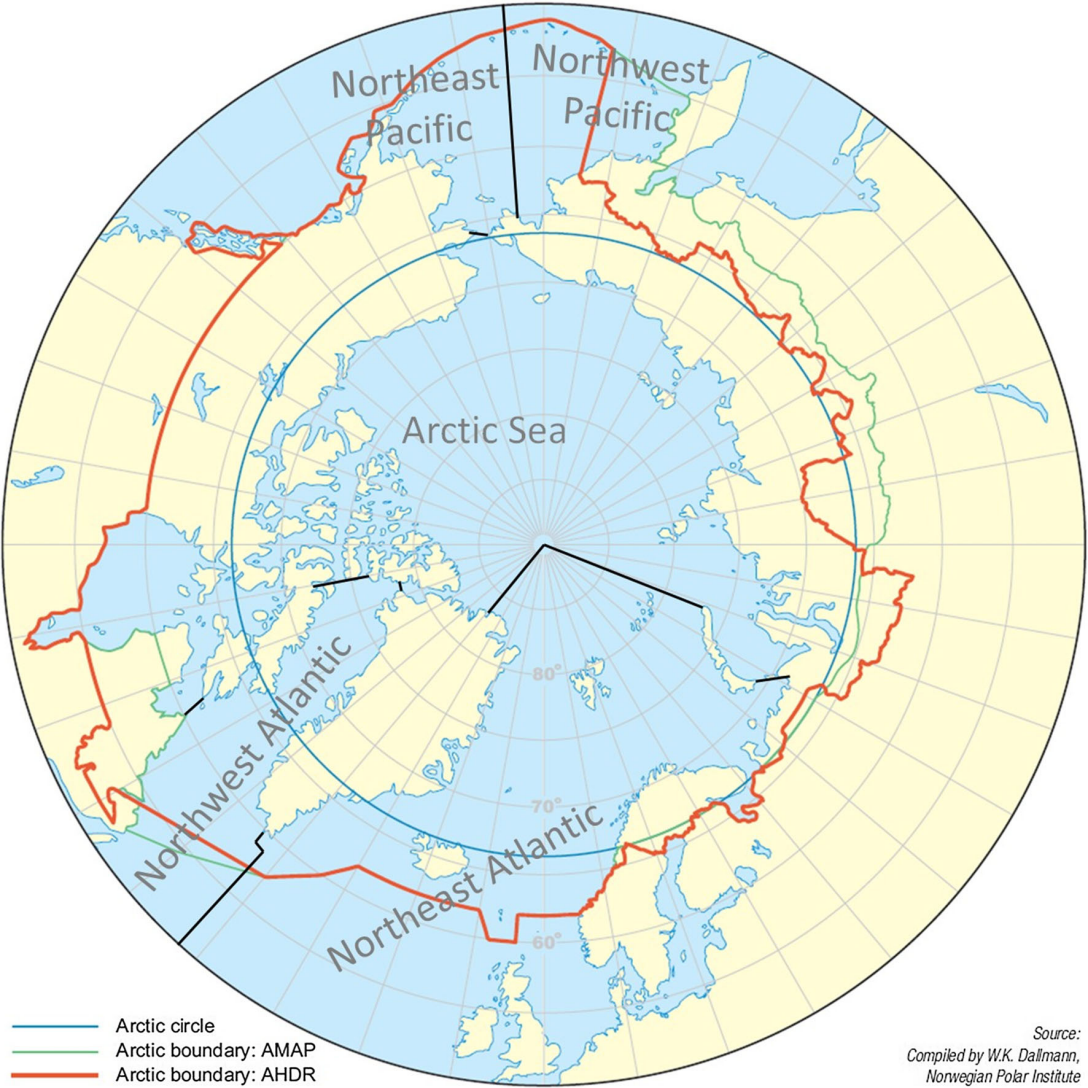
Different areal definitions of the Arctic and FAO major Fishing areas (Source: Young and Einarsson 2004; IPPC 2013)

**Fig. 2:**
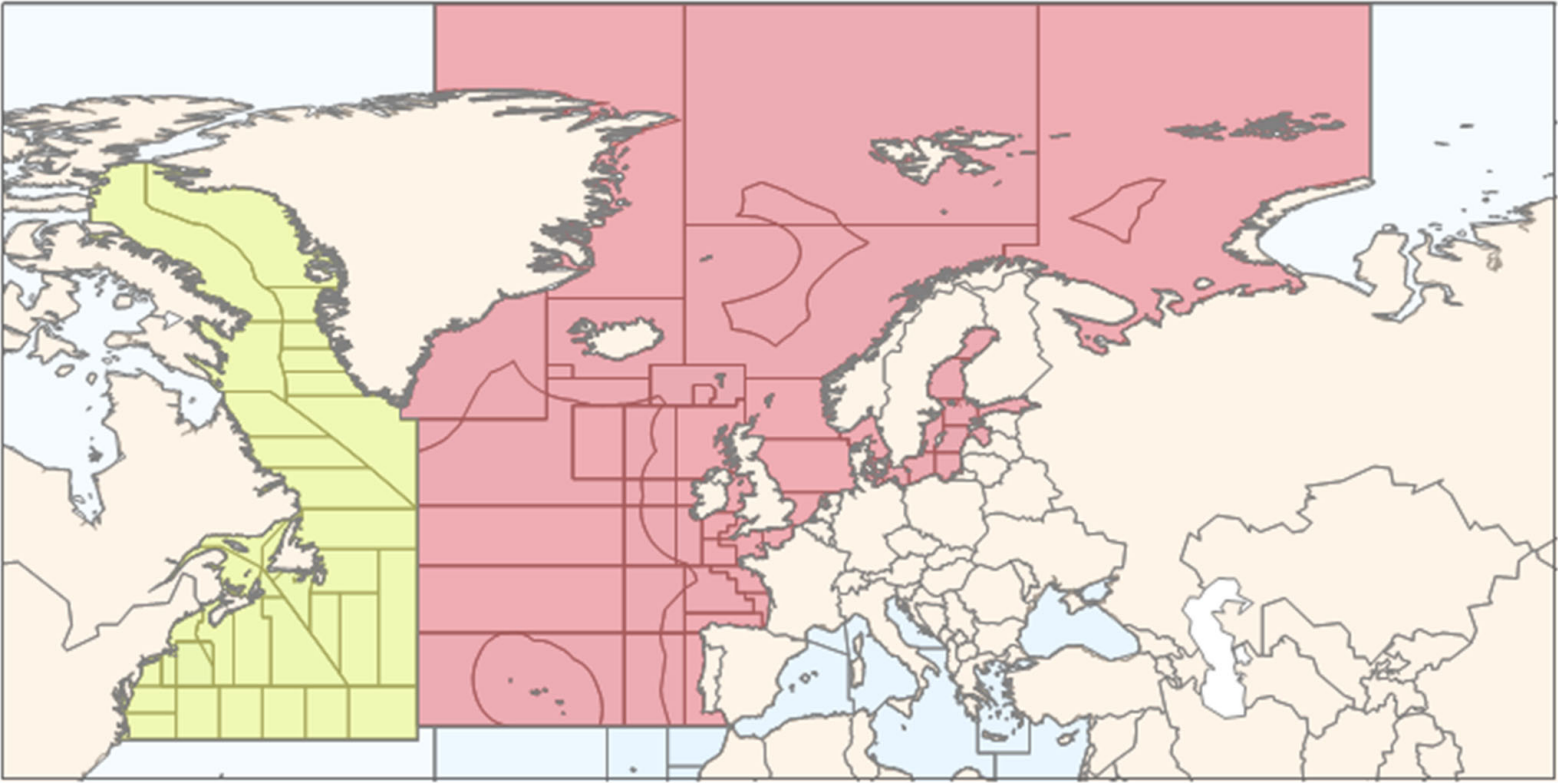
FAO fishing regions: Northeast Atlantic, area 27 (red), and Northwest Atlantic, area 21 (yellow)

In the absence of standard definition (CAFF 2013), we define Arctic capture fisheries to include all catches in marine areas in all Arctic and sub-Arctic waters that lie north of the Arctic Circle (i.e. north of 66°33′N, blue circle in Fig. 1). However, due to reporting structure we also combine FAO statistics (FAO 2014, 2016) with this definition, and relevant FAO fishing areas in the Arctic include the Arctic Sea (Area 18), the Northeast Atlantic (Area 27) and the Northwest Atlantic (Area 21). Not all fishing within these areas is included as it stretches outside the defined Arctic boundary. The Arctic Sea (Area 18) includes Beaufort and Chukchi seas, Hudson Bay, Kara Sea, East Siberian and Laptev seas. The Northeast Atlantic area above the Arctic Circle (subareas I, II, V and XIV of Area 27, Fig. 2) includes the Barents Sea, the Norwegian Sea, Svalbard, the Bear Island, Northeast Greenland, Iceland and the Faroese Grounds, while the Northwest Atlantic area above the Arctic Circle (subareas 0A, 1A and 1B of FAO area 21, Fig. 2) includes part of Baffin Bay and Davis Strait. The Bering Strait being south of the Arctic Circle, we exclude fisheries in Northeast and Northwest Pacific (FAO area 67). In this respect, our definition of the Arctic fisheries area deviates from, for instance, Christensen et al. (2014) in that we exclude the Bering Sea and the Hudson Bay Complex.

This paper only to some extent discusses areas situated just outside of the defined Arctic boundary. To predict possible expansions or relocation of current activity or species migration into the Arctic under climatic change, a more careful consideration of such dynamic would be needed. Aquaculture is strictly limited in the Arctic and focus is here only on marine industrial farming and dominating production nations.

## Global seafood portfolio: the role of the arctic

When narrowing Arctic fisheries to the area above the Arctic Circle, the Barents Sea becomes the most important area regarding catch size. This is one of the most productive ocean areas worldwide. Capture fisheries in the FAO Areas 18, 21 and 27 constituted about 13% of the total world catch in 2014 (Table 1). The Northeast Atlantic is by far the most important area—representing over 10% of global catches. However, both Areas 21 and 27 expand far south of the Arctic Circle. Detailed figures from 2012 show that the share of world marine catches caught in Arctic waters—following our definition—constituted about 6% (4.5 million tonnes), of which 96% were caught in the Northeast Atlantic (Isaksen 2015). Meanwhile, Icelandic waters and the Northwest Atlantic waters (including Greenland and Canadian waters) also have high fishing activity.

**Table 1:** Capture fisheries in tonnes and per cent of world volumes by areas in 2014. The table includes marine fishes, marine crustaceans and Atlantic salmon. Source: http://www.fao.org/fishery/about/en (FAO, accessed 24 October 2016)

FAO area n^o^	Area	Quantity (tonnes)	% of world catch
18	Arctic Sea	1	0.0
21	Atlantic, Northwest	1 275 793	1.6
27	Atlantic, Northeast	8 404 272	10.7
31	Atlantic, Western Central	1 069 779	1.4
34	Atlantic, Eastern Central	4 273 734	5.4
37	Mediterranean and Black Sea	1 259 177	1.6
41	Atlantic, Southwest	1 462 539	1.9
47	Atlantic, Southeast	1 564 711	2.0
48	Atlantic, Antarctic	296 573	0.4
51	Indian Ocean, Western	4 985 455	6.3
57	Indian Ocean, Eastern	7 731 971	9.8
58	Indian Ocean, Antarctic	11 806	0.0
61	Pacific, Northwest	21 843 310	27.8
67	Pacific, Northeast	2 756 673	3.5
71	Pacific, Western Central	13 439 292	17.1
77	Pacific, Eastern Central	1 831 763	2.3
81	Pacific, Southwest	517 198	0.7
87	Pacific, Southeast	5 862 906	7.5
88	Pacific, Antarctic	3 501	0.0
	Total	78 592 468	100.0

The dominating Arctic fishing areas are the Norwegian Sea, the Icelandic Grounds and the Barents Sea (FAO Area 27, Divisions II, Va and I, respectively). Catches from East and West of Greenland (including the Arctic Sea) are marginal in comparison; however, fishing in these areas constitutes important livelihoods for many small-scale operators. North-eastern Pacific fisheries take place south of the Arctic Circle and their numbers are included just as a reference as important Alaska Pollock (*Theragra chalcogramma*) fishery takes place in this area (Table 1).

Fishery is one of the most important industries in the Arctic representing large shares of gross domestic product (GDP) in some countries. For local communities fishing, fish processing and/or fish farming can be even more important. Thus, local communities, regions and nations’ degree of dependency on the fishing or associated activities are important, and since fish production volumes are the most available (and reliable) data at hand, this is usually used as a proxy for importance.

**Fig. 3:**
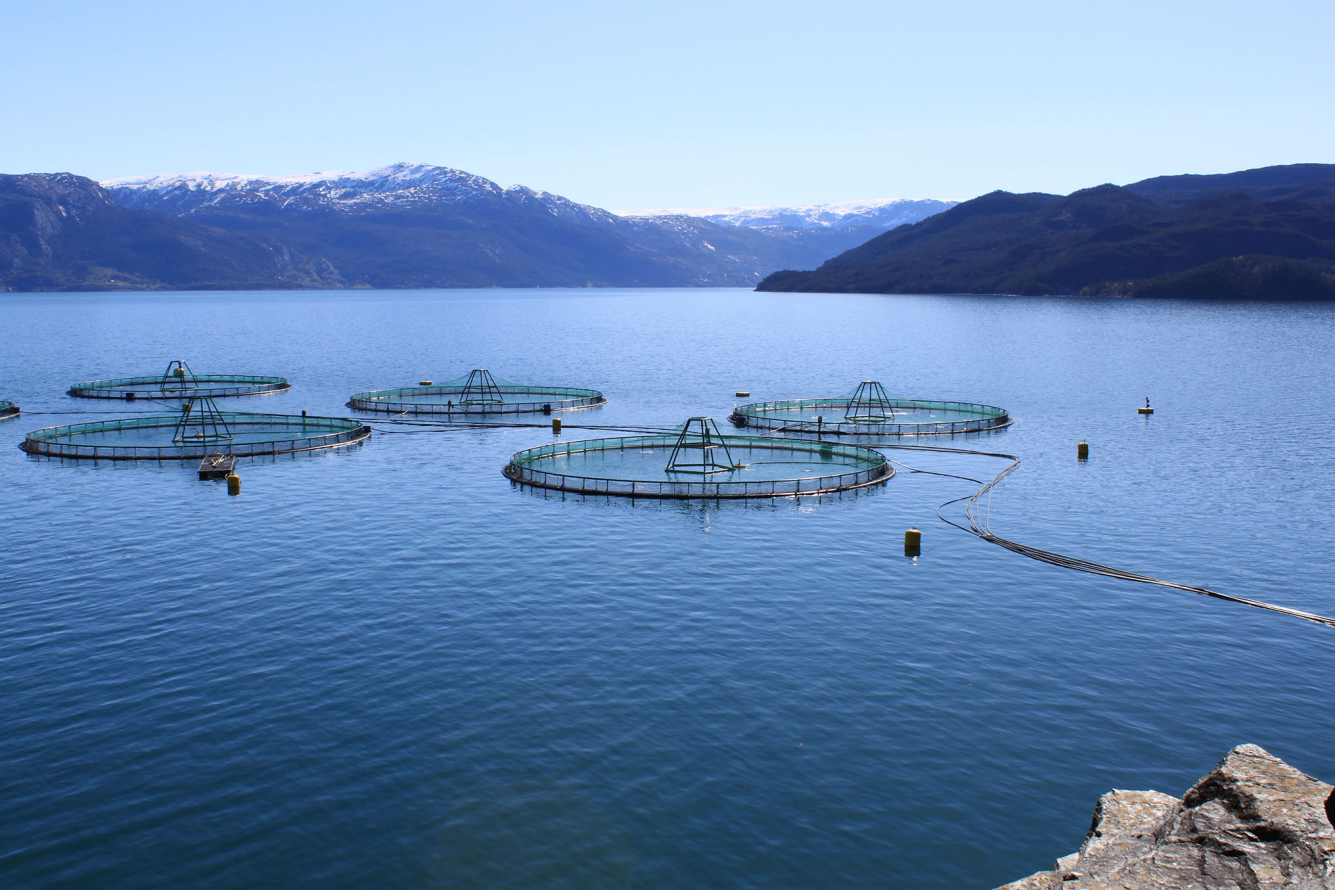
Modern salmon farming cages from the coast of Norway, Photo: R. Lilleholt/Nofima

Fisheries have historically been the main reason for settlement in many peripheral Arctic coastal areas. Arctic aquaculture has the last two decades grown significantly and is today dominated by Norwegian Atlantic salmon farming. Arctic aquaculture only constitutes a small share of world aquaculture production volumes (~ 2%) but its specific contribution to global marine aquaculture production is important (ca. 25%) (FAO 2016) and its economic contribution is even higher. In Norway, fisheries and aquaculture industries contribute only 1% of GDP and 1% of employment. However, fish is the second most important export product (after oil) with nearly 7800 million € in 2014—where more than half the value stems from farmed salmon (Statistics Norway 2015; Norwegian Seafood Council 2015) (Fig. 3).

### Capture fisheries

Volumes of catches and targeted species from Arctic fisheries are presented in Table 2. Catches in Area 18 are small and limited to Russian fisheries in the White and Kara seas (Christensen et al. 2014). The major fishing countries in Area 21 are the USA, Canada and Greenland, while Norway, Iceland and Russia are the main fishing nations in Area 27. The USA, Canada and Russia totally dominate catches in Area 67 (FAO 2016).

**Table 2:** Main economic actors, targeted species and volumes landed from Northeast Atlantic (FAO area 27) fisheries in 2014. Volumes in tonnes. Source: FAO (2016)

Country	Volume	Share (%)	Species	Volume	Share (%)
Norway	2 133 576	25.4	Herrings, Cod, Herring	1 966 681	23.4
Iceland	1 075 639	12.8	Various pelagics	1 880 763	22.4
Russia	993 083	11.8	Cod	1 322 265	15.7
Denmark	693 096	8.2	Blue whiting	1 158 005	13.8
The United Kingdom	675 026	8.0	Saithe	280 925	3.3
Faroe islands	533 380	6.3	Haddock	267 052	3.2
Spain	346 272	4.1	Flounders	262 025	3.1
France	310 045	3.7	Crustaceans	226 638	2.7
Others^a^	1 644 156	19.6	Others	1 039 919	12.4
Total	8 404 272	100.0	Total	8 404 272	100.0

^a^Other nations and catch which are not registered on the listed nations

The main targeted species in the northwest Atlantic (Area 21) are capelin (*Mallotus villosus*), Atlantic herring (*Clupea harengus*), Atlantic cod (*Gadus morhua*), blue whiting (*Micromesistius poutassou*), Greenland halibut (*Reinhardtius hippoglossoides*), queen crab (*Chionoecetes opilio*), deepwater redfish (*Sebastes mentella*) and northern prawn (*Pandalus borealis*) (Lam et al. 2014; FAO 2016). These fishery resources are under stress from past and current exploitation (about 35% of stocks were estimated to be depleted in 2008) (FAO 2010). Some of these stocks have recently shown signs of recovery (Eide et al. 2012) but the collapsed cod stock has not yet recovered (Lam et al. 2014; Nogueira et al. 2016).

Annual landings of all species exclusively from the Arctic region (Area 18) are very low, with only 4 tonnes reported in 2014 (Table 1). However, catches around 500 tonnes were reported in 2008 and 2010, by far the largest since 1970 (FAO 2016) although still very low compared to adjacent regions. The polar cod (*Boreogadus saida*) spawning stock was particularly large in 2008 (FAO 2016), which could explain the large catches. Polar cod stocks experience large variations in this region (Eide et al. 2012). Other Arctic species (stocks that appear only in ice-laden waters and spawn at below-zero temperatures) targeted in Arctic Sea Russian fisheries are navaga (*Eleginus nawaga*) and Arctic flounder (*Liopsetta glacialis*) according to Christensen et al. (2014). The main fishing activity taking place in these areas is inland “*small*-*scale subsistence fisheries among indigenous people (…) mostly* [for] *freshwater and diadromous fishes*” (Christensen et al. 2014, p. 354).

Norway is the dominant fishing nation in the Northeast Atlantic but many countries have active fisheries in this area (Table 2). The main species landed in 2014 were herring, cod and haddock (*Melanogrammus aeglefinus*), together contributing to almost 60% of total catch. The capelin stock was not exploited in 2014 while it represented the largest catch 2 years before (FAO 2016). This large variation illustrates the combined effects of a fluctuating ecosystem and the management system in place. When the capelin stock is below a critical level, the management system prioritises leaving the capelin in the sea as prey for cod instead of catching it (Eide et al. 2012).

The International Council for Exploration of the Seas (ICES) advises on total allowable catch (TAC), which are then set by governments. The TAC advice for Norwegian spring spawning herring (in areas I, II, V, IVa and XIVa) in 2015 was 283 000 tonnes compared to 1 687 000 tonnes in 2009 (ICES 2016). The Northeast Atlantic mackerel (*Scomber scombrus*) has a larger (and increasing) distribution area, and coastal states have not reached a final agreement on the allocation of quotas since 2007. For that stock, the advice went from 349 000–456 000 tonnes in 2008 to 927 000–1 011 000 tonnes in 2014. ICES estimated that 1 396 000 tonnes were landed in 2014 (ICES 2016).

While the fleets from the different nations all target the same common resource, they typically have different structures, with different boat sizes and gear types—often resulting from national institutional constraints. The industry is rather dynamic; for example, in 2013 a new fishery developed along the Eastern Greenland coast targeting mackerel that had recently reached further north in this region and all the way to the Spitsbergen fjords (Jansen et al. 2016). Also, quotas for Northeast Atlantic cod peaked in 2013 (and subsequent years) to the highest catch levels in 40 years (estimated to 966 000 tonnes in 2013). Atlantic herring biomass is decreasing in this area, while mackerel landings have been increasing since 2005. However, inter-year climate variations are large. For example, the joint Norwegian Russian Ecosystem survey in the Barents Sea could not survey the Spitsbergen region from August to October 2014 due to increased ice coverage compared to 2013 (Eriksen 2014).

**Fig. 4:**
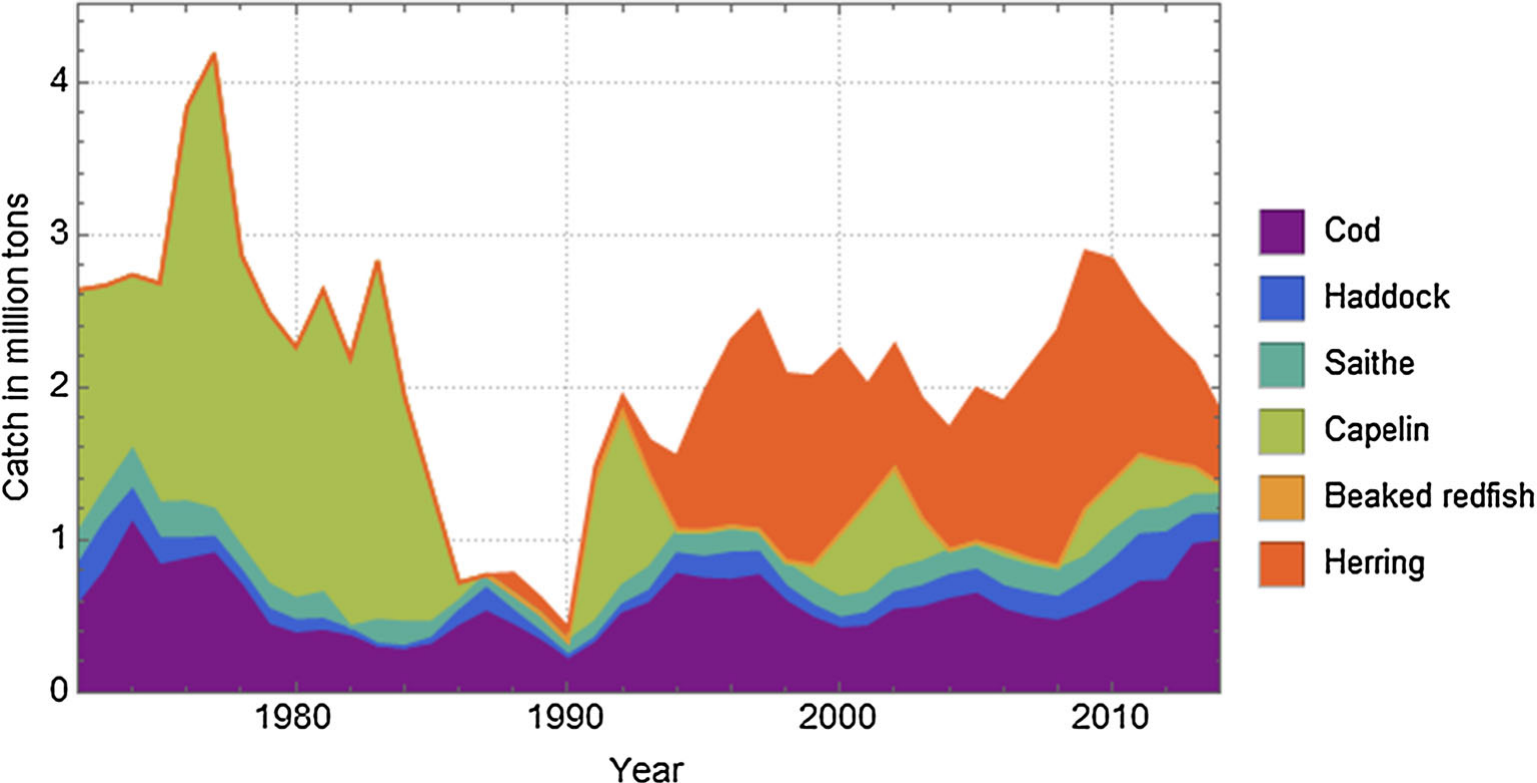
Barents Sea catches of the main targeted species during the period 1972–2014 (Source: Anon. 2015)

The Barents Sea is one of the most productive oceans in the world; the economically most important species are the Northeast Arctic cod, haddock and capelin, the latter being the main prey species of cod. Russia and Norway share these fish stocks. Occasionally large numbers of juveniles of the spring spawning herring (essentially a Norwegian Sea stock) flow into the Barents Sea basin, significantly affecting cod–capelin interaction (Huse et al. 2002). Figure 4 shows catches of the main targeted species during the period in the Barents Sea. The figure reveals some correlation between these stocks. After the spring spawning herring collapsed at the end of the 1960s, a large capelin fishery took off in the Barents Sea. When the herring stock recovered, large quantities of young herring entered the Barents Sea in the mid-1980s. They preyed upon pelagic capelin larvae, thus causing a collapse in the capelin stock with a following starvation in the cod stock (Hamre 1994; Tjelmeland and Bogstad 1998; Gjøsæter et al. 2016). In the mid-1990s, another inflow of young herring caused a similar decline in the capelin stock but this time new management strategies quickly closed the capelin fishery preventing additional fishing pressure on the capelin stock (Fig. 4) (Gjøsæter et al. 2016).

### Norwegian fisheries^1^

Norwegian fisheries are diverse and like in other countries they have experienced considerable changes recently. From 2000 to 2014, the number of registered fishing vessels decreased from 13 000 to 6000. About 80% of the fleet in 2014 consisted of small vessels below 11 metres (Fiskeridirektoratet 2015). However, this vessel group experienced the largest reduction in the number of vessels (approx. − 56%). The group of larger offshore vessels (cod trawlers, purse seiners, etc.), above 28 m, comprised about 250 vessels in 2014—a 30% reduction from 380 in 2000. The larger vessels were responsible for the largest share of the total catch (80% in 2014), while the share of the smaller vessels was only about 6% (Fiskeridirektoratet 2016). However, smaller vessels had a larger share of catch value (11% in 2014) because these targeted more high-price species (mainly cod), while larger vessels targeted mainly low-valued pelagic species. The share of catch value for the larger vessels was 70%. Of the 6100 registered vessels in 2013, only 5200 were registered landings and 4100 with a catch value for 2013 above € 6400. The Norwegian Directorate of Fisheries included 1748 vessels in a profitability study for 2014 and showed that these vessels were responsible for 91.5% of total catch value that year (about 1727 million €) (Fiskeridirektoratet 2016). Cod was the most important single species in Norwegian fisheries, constituting 20% of catch and 32% catch value, in 2014, compared to catch value for mackerel (14%), herring (13%), saithe (*Pollachius virens*, 9%) and haddock (8%). Between 2000 and 2014, the number of registered fishermen in Norway decreased from 20 000 to 11 300 (− 44%).

### Icelandic fisheries^2^

The Icelandic fishing fleet consists of 1700 fishing vessels 2013, 15% fewer than in 2000^3^. Many are open vessels and smaller decked vessels, in addition to trawlers and larger decked vessels (Statistics Iceland 2015). During that period, open vessels became 22% fewer (from 1100 to 860), medium-sized decked vessels (trawlers incl., 100–499 gross tonnage (GT), 500–999 GT and 1000–1499 GT) decreased by 40–50% (from 270 to 160 vessels), while the number of smaller decked vessels (less than 100 GT) increased with 7% (from 600 to 650), and the largest vessels (above 1500 GT) increased from 12 to 26. Trawlers and large decked vessels target both demersal and pelagic species. However, they may increasingly target pelagic species as prices for pelagic fish increase, due to increased demand for human consumption instead of fishmeal and fish oil. Pelagic catches already dominate the volume (64%) and constitute about 30% of catch value, while demersal species amount to one third of the volume but 61% of value. The dominant species in Icelandic fisheries are (with share of landings in brackets) capelin (32%), cod (17%), herring (12%) and blue whiting (8%). Cod alone represents 10% of landings’ value. The 1500 open and decked vessels below 100 GT landed 4% of total volume in Icelandic fisheries in 2013 (1.4 million tonnes), which constitutes 8% of the total value (153 billion ISK ≈ 942 million €). At the same time, the 75 largest vessels’ (above 1000 GT) share of volume was 68% and, correspondingly, 50% of the catch value. In the period 2000–2013, the number of vessels was significantly reduced from 6100 to 3600.

### Northwest Russian fisheries

The Northwest Russian fishing fleet is more homogeneous than other Arctic fishery nations. Under the Soviet era, industrialisation and large-scale operations led to a focus on large trawlers supplying large seafood processing units on land. Also, with limited fish resources near the coast, a coastal fleet never developed in Northwest Russia (like it did in Norway, Iceland and Greenland). The industrial fleet is located mainly in the Murmansk and also in Arkhangelsk Oblast. Murmansk Regional Government (MRG 2015) reports the Murmansk industrial fishing fleet to have 207 vessels, including 11 extra-large vessels, 11 large vessels, 117 medium-sized vessels and 68 small vessels.^4^ In addition, there are about 100 vessels of different types active in coastal fisheries^5^ responsible for landing about 22 000 tonnes of seafood—about 3% of total landings in Murmansk region in 2013 (i.e. 700 000 tonnes; MRG 2015) compared to approximately 450 vessels 10 years ago (Vilhjálmsson and Hoel 2004).

Russian official catch statistics^6^ report a total Russian marine capture of 4 million tonnes in 2013, of which 25% (1 million tonnes) were caught in the Northeast Atlantic, 83% of which were caught in Arctic areas (834 000 tonnes).^7^ Cod was the most important species (41% of landings from Arctic areas), and other species were haddock (18%), herring (14%), mackerel (9%) and capelin (8%). Russian landings to Russian ports peaked after 2009 after a decision to remove excessive formalities on documentation of landing operations and stop treating them as taxed imports (FAO 2012). Still today, large shares of Russian Northeast Atlantic catches are landed abroad even though the seafood processing industry in the Murmansk region processes roughly 550 000 tonnes of fish on an annual basis (Moran 2013).

The Murmansk fishery sector’s share of regional GDP is 7%, and the sector employs roughly 7800 persons (MRG 2015). Murmansk was the port in Russia with the largest catch value in 2013 (shipped fish production, with approximately 732 million €). The number of fisheries employees was 6200 in 2012, 41% less than in 2005 (Boboedova 2014). Employment in the fishery sector in the whole Russia was 59 200 in 2013.^8^

### Greenland fisheries

Fishing is Greenland’s primary industry and shrimps (*Pandalus borealis*) are the most important species. Greenland fisheries’ share of GDP was 13.6 and 90% of total export in 2013^9^ and http://www.stat.gl/publ/da/IE/201401/pdf/Udenrigshandel2013.pdf ). In 2013, the fishing fleet consisted of 384 vessels, of which 193 were below 10 m, 149 were 10–20 m, 19 were 20–30 m and 23 were larger than 30 m in length. In addition, Greenland fisheries sector also had 185 snowmobiles, 602 dog sledges and 1422 jolly boats, mainly located in Northwest Greenland (Qaasuitsup) and with permit to fish and land fish (and also marine mammals). These “vessels” had a share of 29% of total Greenland landings value (about 117 million € in 2013). Greenland’s total catch in 2013 amounted to 170 000 tonnes, 70% from within their own exclusive economic zone (EEZ). The most important species (in volume) were mackerel (31%) caught in East Greenland waters and ICES areas XIV a/b, shrimp (25%) and capelin (16%) caught in Icelandic waters and Greenland halibut (6%). In value terms, shrimp was by far the most important species (63% of 161 million € in total), then Greenland halibut (12%) and cod (7%). Shrimp trawlers were either larger offshore trawlers or inshore trawlers. The former operated outside three nautical miles from the baseline and in open waters and had an obligation to land 25% of its catch to land-based production (leaving 75% to be on-board processed and exported). The inshore trawlers had an obligation to land 100% for land-based production. Greenlandic shrimp quotas were divided between offshore and inshore trawlers in a 57/43 percentage distribution. In addition to fish and crustacean, Greenlandic hunting landed 51 000 sealskins (from a total catch of 105 000 seals) and 3300 whales (of which 70 percent harbour porpoise whales—*Phocoena phocoena*) in 2013.

Of the most important species (cod, crab, shrimp and halibut), approximately 116 000 tonnes were caught in NAFO areas 1a (Baffin Bay) and 1b (Davies’ Strait), while 8 000 tonnes were caught in ICES area XIV (a and b) and other ICES areas. Overall, approximately 125 000 tonnes of Greenland’s catch were caught in Arctic waters (55%). Catches in Arctic Northeast Atlantic waters are unclear (ICES catch statistics for 2012 report 39 000 tonnes but EuroStat has no records). Total employment in the Greenland fisheries sector (fish processing industry excl.) was roughly 3550 in 2013—approximately 13% of Greenland’s labour force.

## Aquaculture

**Fig. 5:**
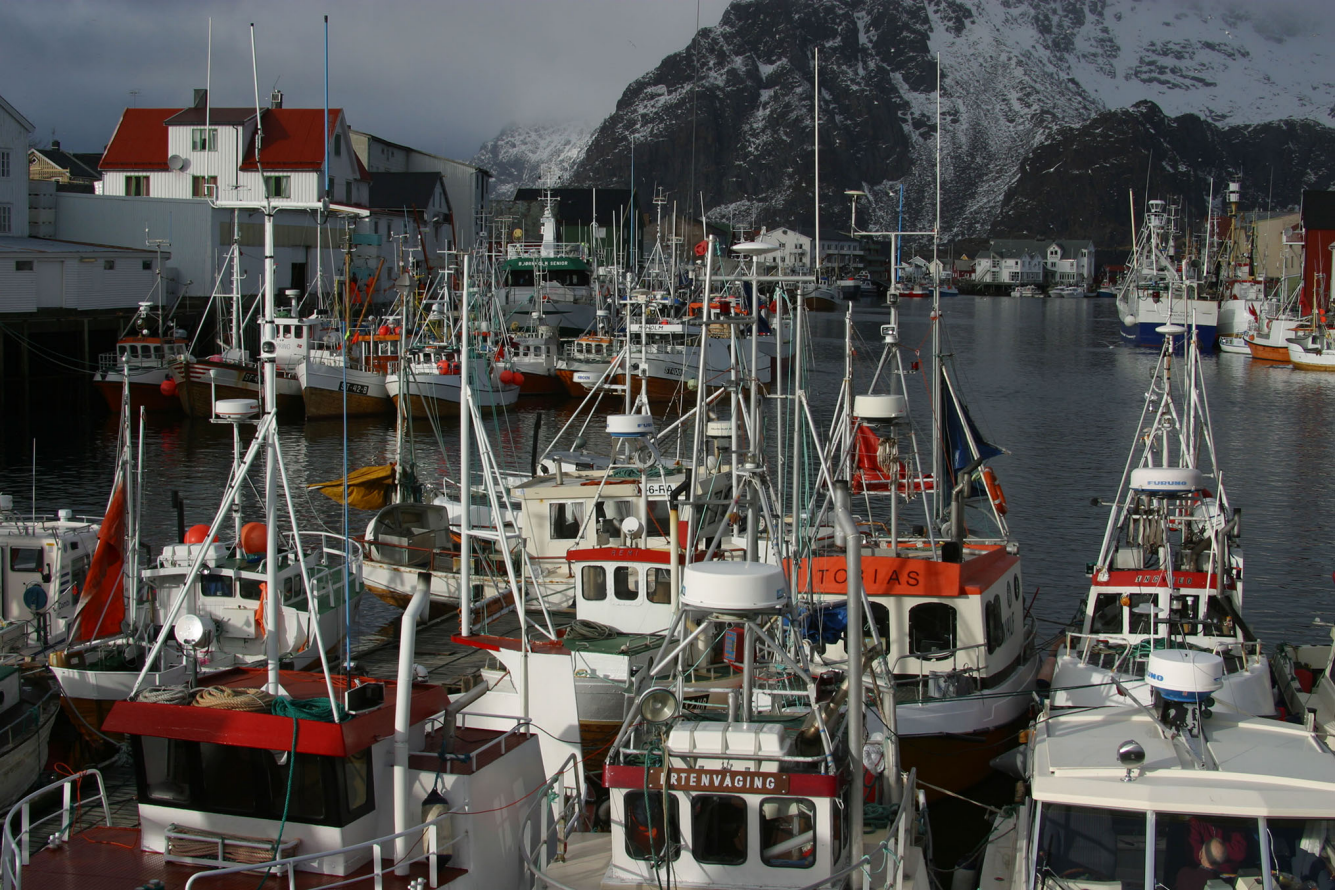
Coastal fishing vessels gathered in Henningsvær (Lofoten) during the traditional cod fishery, winter 2011, Photo: Frank Gregersen/Nofima

**Fig. 6:**
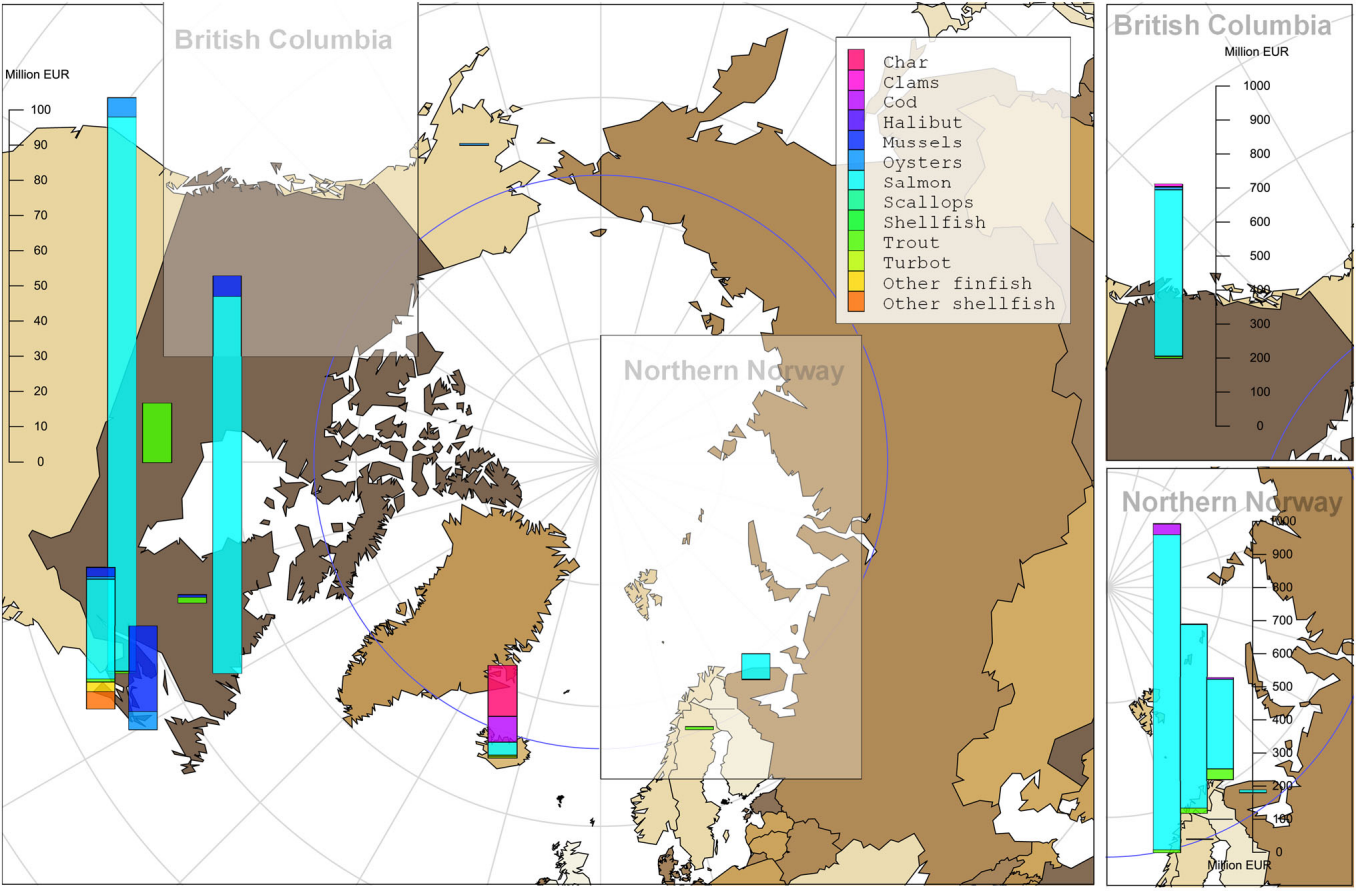
Aquaculture production (values) in the Arctic and selected surrounding areas by location, species and value in 2014. Norway and British Columbia are shown separately and for Norway the three main producing counties (Nordland, Troms and Finnmark, Finnmark with the most northerly production) are shown. (Source: National aquaculture statistics and FAO 2016)

Aquaculture is an important economic activity in some concentrated parts of the Arctic region (Fig. 5). Total Arctic aquaculture production constitutes about 2% of both global production volumes and global values of fish and shellfish (FAO 2014). Figure 6 provides an overview of the production and geographic species distribution of aquaculture in the Arctic region and some surrounding areas. Norway accounts for 93% of the total value of Arctic aquaculture, which mostly consists of salmonid production and is a significant contributor to rural economies and employment (Andreassen and Robertsen 2014), although its benefits to local communities has been argued to diminish during the last two decades (Isaksen and Mikkelsen 2012). Norway is also the main producer of rainbow trout (*Oncorhynchus mykiss*). Iceland mainly produces Atlantic salmon, rainbow trout and Arctic char (*Salvelinus alpinus*), while Russia produces primarily salmon. In Finland and Sweden, small volumes of freshwater species dominate the production. Some mussels (*Mytilus edulis*) are produced in areas close to the Arctic such as Newfoundland and in the southern parts of Alaska. Fish farming is currently prohibited in Alaska. In the Canadian provinces south of Newfoundland, both salmon and mussels are farmed.

### Norway

Norway is the world’s largest producer of Atlantic salmon and also has a significant production of rainbow trout and a smaller production of several other marine and freshwater species. A considerable part of this production takes place in the Arctic region. Table 3 illustrates production data from the northernmost three counties. In 2014, salmon constituted 98% of the total value (2200 million USD) and trout constituted 1.5%. The southernmost county of the three, Nordland, is the dominating producer.

**Table 3:** Values of Norwegian aquaculture production by county in 2010 (1000 EUR) (Source: Norwegian Directorate of Fisheries)

	Nordland	Troms	Finnmark	Total
Salmon	1 114 337	714 310	367 277	2 195 924
Rainbow trout	17 679	15 947		33 626
Other finfish	3556			3556

Salmon farming was introduced in the Norwegian Arctic around 1970 and grew rapidly after 1994. Salmon dominated during the whole period and the value of production accelerated after 2000. Other species were introduced, but have not experienced the same growth as salmon despite considerable research and development investments for farming halibut and cod for example. Halibut was primarily in focus during the early 1990s and cod in focus during the latest part of 1990s. Arctic charr was early in 1900 a pioneering aquaculture species in Norway, but farming north of the Arctic Circle developed first during the 1980s (Sæther et al. 2016). Farming of blue mussels was rather unsuccessful too due to toxic algae, among other reasons (Winther et al. 2010).

### Iceland

Only a limited part of Iceland’s coastline is protected against waves and suitable for aquaculture, and hence production is relatively small. Icelandic aquaculture has gone through several phases, with rapid growth in the 1980s followed by a period of stable production in the 1990s and a rapid decline in the mid-2000s when salmon production declined from about 7000 to about 500 tonnes. Arctic charr have had a relatively steady growth during the whole period and cod aquaculture started to develop first in early 2000. Arctic charr constitute about 50% of total value (Table 4). Atlantic cod and Atlantic salmon and some minor production of Atlantic halibut (*Hippoglossus hippoglossus*), turbot (*Scophthalmus maximus*), trout and blue mussels constitute the rest of the production. A large share of the overall production originates from land-based systems.

**Table 4:** Values of Icelandic aquaculture production in 2014 (1000 EUR, Source: FAO FishStat)

	Land-based
Arctic char	27 288
Atlantic cod	992
Atlantic salmon	19 032
Rainbow trout	3196
Blue mussel	144
Total	50 652

**Box 1:** Food production and food security in the Arctic

Food production and food security in the Arctic is complex and does not only encompass production and local people’s access to nutrition (The Alaskan Inuit Food Security Conceptual Framework, Inuit Circumpolar Council-Alaska (ICC-AK) 2015). Ongoing and emerging climate change also poses a threat to existing food systems and food security by affecting indigenous peoples’ access to wildlife (Gunn et al. 2006; Tesar 2007; Heleniak 2014). For example, climate change alters migratory patterns of Arctic animals and i.e. Inuit hunters are struggling to adapt. Thus, the implications for traditional hunting, fishing, and gathering activities will have crucial local economic and dietary importance, and impact on social and cultural identities (Nuttall et al. 2005). Imported foods have increasingly replaced local foods (Kuhnlein 1992; Wein and Freeman 1992) resulting in higher frequency of obesity, diabetes and heart diseases from increased intake of carbohydrates and saturated fats (Blanchet et al. 2000; Kuhnlein et al. 2000; Van Oost dam et al. 2005). In addition, marine mammals such as beluga and seals contain higher levels of contaminants, which impacts severely on indigenous peoples’ health. The relation with climate change is uncertain (NCP 2013). Warming may also increase opportunities for local food production in some regions as growing periods becomes more favourable. This means that conditions for crops and livestock farming will improve. A longer open-water season will make transportation more viable throughout the year, something that may reduce costs for local agriculture production inputs like e.g. seeds, fertilizers, fuel, etc. (IPCC 2001). Poor soil, unpredictable climate, and supply costs will still remain a challenge for the growth of the Arctic agricultural sector. In addition, although climate change resulted in warmer summers, they are also drier with potential negative implications for growth. Emergence of new shipping routes resulting from changed ice conditions may also influence Arctic people in multiple ways, however, resulting impacts on e.g. the fishing sector is difficult to foresee. Photo: Hunting and gathering have historically been the primary methods of supplying food for many local coastal communities in the Arctic. Climate change has influenced migratory patterns of Arctic animals forcing Inuit hunters to adapt. This adaptation has costs in the form of time, money and increased risks. (Photo: Wanny Woldstad, Photographer Unknown–Tromsø University Museum	

Arctic charr constitute over 50% of total production (Table 4), with Atlantic salmon constituting a large fraction of the remaining half. Rainbow trout, Atlantic cod and some minor production of mussels constitute the rest. A large share of the overall production originates from land-based systems.

## Climate change: challenges for governance in the arctic

### Fisheries

#### Spatial distributions and ecosystem effects of environmental changes

Climate change can affect the main drivers causing distributional changes in the productivity of an ecosystem (Brander 2010). Natural fluctuations in the physical and biological environment strongly affect sub-Arctic marine fish populations (Dippner and Ottersen 2001; Godø 2003). However, since today’s climate models do not include scenarios on ocean temperatures, water mass mixing and upwelling, which fisheries typically depend upon (through primary and secondary production), predictions regarding future fisheries’ response to climate change are of tentative nature (Vílhjálmsson et al. 2005).

Historically (17th and 18th century) claimed increased catches were associated with increased temperatures (Lajus et al. 2005). More specifically, ocean temperature seems correlated with cod stock recruitment and growth (Dippner and Ottersen 2001), suggesting that large-scale atmospheric variability seems to affect recruitment and growth in the Barents Sea ecosystem (Ottersen and Stenseth 2001). Warmer periods seem to favour northern cod populations but at the same time stress southern populations.

While increased temperatures may lead to more northern distribution of cod stocks in the sub-Arctic (Drinkwater 2005), fishing activities have earlier also affected the distribution of cod stocks (Engelhard et al. 2013). Depth may be a constraining factor for benthic species, preventing a further northern expansion north and west of Svalbard (Eide 2014, 2016; Fossheim et al. 2015). However, it is not necessarily a constraining factor for pelagic species. Spatial distribution of capelin tends to follow environmental changes, and a spread northward in the Barents Sea area is probably a response to recent increase in temperature and reduced ice cover (Ingvaldsen and Gjøsæter 2013). Changes in the spatial distribution of capelin may change its condition and fat content due to distributional patterns of zooplankton communities (Orlova et al. 2010). The overall ecosystem consequences from this are complex and difficult to predict at large scale.

Christiansen et al. (2014) point to the potential degradation of Arctic species and ecosystems due to inevitable bycatch and bottom trawling harming the seabed, as the ice retracts and commercial fish and fisheries displace polewards. These understudied species are particularly dependent on a precautionary approach in fisheries management practices, since uncertainty levels are high and so also the risk for stocks to collapse. However, according to Vílhjálmsson et al. (2005) it is likely that a moderate warming will improve the conditions for the most important fish stocks in the Arctic, like cod and herring, but it will also contribute to a very different species composition in some ecosystems. Hence, commercial fisheries must be adjusted, which might call for renewed fisheries negotiations for fishing rights and TACs (total allowable catch) among coastal states. As a consequence, the effect of climate change on fish stocks might originate to a larger extent from policies and their enforcements than from climate warming itself if it is moderate.

Temperatures may not be the main explanatory factor for changes in the distribution of pelagic fish populations in the Arctic (Pacariz et al. 2016). A case study of the North-eastern Atlantic mackerel stock concludes that seafloor topography, ocean currents and nutrient limitations are other important explanatory factors for pelagic species. Ocean currents and nutrient densities are closely connected to the topography of the seafloor, which is not affected by climate change. Hence, climate-induced spatial changes of fish stocks and ecosystem may be less than expected when focusing only on temperature. For benthic species like the Northeast Arctic cod stock, centres of gravity of monthly spatial distributions of biomasses do not seem to change significantly due to climate change in the next 50 years (Eide 2016).

#### Acidification, sea level rise and storms

The world oceans have absorbed approximately 30% of the atmospheric CO_2_ deriving from human activities, leading to reduced pH values in ocean water but regional differences are substantial (Hoegh-Guldberg et al. 2014). Acidification therefore represents a concern but knowledge about biological and environmental impacts is poor at this stage (Bates and Mathis 2009; Hoegh-Guldberg et al. 2014). Climate change is expected to increase intensity and frequency of storms in some ocean areas. It is however not certain that this represents a significant change or may be explained within the long-term patterns of variability (Hoegh-Guldberg et al. 2014).

#### Management challenges

The large cod stock in the Barents Sea being distributed over a vast area could be the result of the combined effect of successful management and increased productivity resulting from favourable climate factors (Kjesbu et al. 2014). Studies of the Icelandic cod fishery (Mazzi 2005) indicate that fisheries management indeed affects the prediction value of climate processes in explaining changes in recruitment and growth. Harvest control rules and precautionary approaches to management could represent more useful stock management strategies compared to using complex ecosystem models including climate variables. Indeed, the complexity of the climate system and lack of knowledge regarding model specification and parameterization effectively hinder improving management decisions (Punt et al. 2014; Kvamsdal et al. 2016).

Management of many shared sub-Arctic fish stock builds on agreed shares of the TAC. Redistribution of common stocks due to climate change or other reasons potentially threaten existing agreements and also include new stakeholders and nations that previously were not exploiting these stocks. For example, in the North-eastern mackerel fishery, the emergence of an Icelandic mackerel fishery undermined previous agreements (Jansen et al. 2016). The risk of management collapse was not enough to bring the partners together. However, despite the lack of formal agreements, the expected serious overfishing did not occur. Instead, quasi-cooperation contributed to hinder overfishing (Hannesson 2014). Although falling somewhat short compared with full management cooperation, the partners shared the basic principles of fisheries management and eventually acted according to their ideas about responsible fisheries.

#### Markets (factor markets and consumer markets)

Climate change may also indirectly influence demand for fish products, factor markets for production of fishing effort and the processing industry. Increased public awareness about climate change could put a price premium on products with less negative climate impact, i.e. products in which the production process generates less greenhouse gas emissions compared to others. Hence, demand for seafood could skew towards products that are harvested, processed and distributed in a more climate-friendly manner unless more responsible fisheries preventing bycatch also happen to be more energy intensive creating conflicting environmental objectives. Consumer power is organised to some degree through organisations that certify seafood based on sustainability and/or lesser climate impact (like ecolabelling and seafood sustainability standards such as Marine Stewardship Council, Aquaculture Stewardship Council, Friends of the Sea, WWF, Seafood Watch, KRAV and others). Price premiums have been observed on labelled seafood (Roheim et al. 2011) but it is also criticised for being a necessary market signal in business-to-business marketing, not necessarily towards the customers (Roheim and Sutinen 2006; Washington and Ababouch 2011; Nøstvold et al. 2013). Other studies have observed price premiums for long-line caught seafood in the UK market (Sogn-Grundvåg et al. 2013), but the price premium can just as well come from the extra quality delivered by this fishing gear rather than the environmental concern.

Fishing industries worldwide, including all major fishing fleets in the Northeast Atlantic, benefit from heavily subsidised fuel prices (Tyedmers et al. 2005; Martini, 2012; Isaksen et al. 2015; Waldo et al. 2016). The fleets’ shares of national emissions are relatively modest (except for Greenland); however, abolishing fuel subsidy schemes could reduce emissions and potentially alter operations and fleet composition. However, since high-water fisheries in these areas often take place in international waters, international cooperation is necessary to avoid leakage (i.e. fuel bunkering abroad or in international waters, with the potential landings going the same way). It is, however, important to compare environmental performance (e.g. GHG emission) of fishing with production of animal proteins from land systems. Captured fish (and farmed fish) can offer more sustainable alternatives (Troell et al. 2014).

### Aquaculture

Abiotic environmental conditions like water temperature, salinity, oxygen content and water quality (Mydlarz et al. 2006) and physical processes associated with waves, currents, tides, ice and river (Troell et al. 2009; Callaway et al. 2012) influence aquaculture conditions. Most studies of climatic effects on aquaculture discard indirect effects that potentially can impact aquaculture, e.g. changes in agriculture production, financial markets, demographic structures and capture fisheries (Handisyde et al. 2006; Cochrane et al. 2009). However, the impacts of environmental changes on aquaculture often result from a chain of effects, hence making it difficult to identify clear causative links (De Silva and Soto 2009). The impacts of temperature and increase in extreme weather events on fish growth are examples of direct effects. Indirect effects include increased risks for diseases or pathogen infections due to higher temperature and changes in input factors, especially those linked to resources from capture fisheries and agriculture through feeds (Troell et al. 2014; Cao et al. 2015) and energy inputs.

Air and water temperature, sea level change, water current, wind and waves, ice and salinity are the climatic drivers most likely to impact on aquaculture (Handisyde et al. 2006). Changes in extremes, like storms and temperature extremes, can influence physiology (growth, reproduction and disease outbreaks), ecology (organic cycles and parasites) and farm operation (sites and technology).

#### Growth and productivity

Sea temperature directly influences metabolism and growth. Most fish have an optimal temperature for growth so deviation from this optimum will restrict growth. Salmonid have a relatively narrow range of temperature for optimal growth (Ficke et al. 2007). Hence, present optimal conditions for open sea cage salmon farming in Norway lie between 62° and 64°N latitude. Further south, summer temperatures are higher than optimum, and further north, temperatures are too low throughout the year. Increased sea temperatures will generally move this optimum zone further north. For fish farms in colder locations than optimal, production can increase with 11–15% per degree increase in temperature (Lorentzen 2008). For farms at optimum or higher temperatures, production will decrease. Salmon farms in the Arctic generally experience lower than optimum temperatures and will likely experience improved productivity. Species like cod and halibut have narrower temperature ranges (Imsland et al. 2000; Levesque et al. 2005) but should respond in a similar way. The temperature optimum also decreases with increasing size of the fish, which further complicates the predictions of actual impacts from changes in temperature. Farmers currently operating in areas that will experience significant temperature changes can mitigate adverse effects through re-siting/re-establishing their farms in areas with temperature range closer to optimal. Production loss due to temperature changes, relocation costs, property rights, permits and existing infrastructure will influence to what extent relocations occur.

The decrease in oxygen solubility, combined with the higher metabolic rates and oxygen consumption associated with higher temperature, may impact the carrying capacity of a site. However, the farmers plan their stocking densities according to oxygen availability at any time. Hence, locations with insufficient water exchange may have to reduce the density of fish to avoid oxygen depletion that could hamper fish growth.

#### Sea level rise

Several direct and indirect effects of climate change could result in a net sea level rise due to increased water volume at higher temperatures and melting of ice caps in, e.g., Greenland (Parry et al. 2007). Sea level may change between − 20 and + 30 centimetres along the Norwegian coast although these estimates are uncertain (Simpson et al. 2012). Sea level rise within this range is unlikely to significantly impact sea-based aquaculture.

#### Storms

Models predict more frequent and more intense storms in the northeast Atlantic (Leckebusch et al. 2006; Frost et al. 2012), although this shift is suggested to take long time to evolve (Weisse et al. 2005) and is uncertain. Storms can severely impact sea-based farms, while land-based facilities are less exposed. For cage farms at sea, most breakdowns occur during storms due to strong waves and icing. These damage structures and result in fish dying or escaping. Fish escaping to the wild and breeding with native populations can cause hybridization and loss of genetic diversity (Walker et al. 2006). Storm patterns will likely change slowly providing sufficient time for the industry to adapt by strengthening structures (optimising for offshore farming) or moving to less exposed sites. These measures increase costs and moving may be difficult due to lack of optimal or even available sites, thus reducing productivity and profits.

#### Diseases and parasites

Climate predictions indicate longer and more frequent periods of extreme temperatures (IPCC 2013). Higher sea temperatures influence fish growth and disease. Diseases occur in most living organisms and increasingly so in farmed animals because the high biomass concentration in farms provides attractive breeding grounds for pathogens. Temperature extremes close to the fishes’ tolerance levels, combined with oxygen depletion, result in physiological stress and increased vulnerability to diseases. Changes in temperature can also change disease occurrence and spreading patterns in rather unpredictable ways (Gubbins 2006). Pathogens have often shorter generation times at higher temperatures (Duguid et al. 1978). Common diseases in salmon and cod aquaculture include fransicellosis, vibriosis and furunculosis, all associated with high water temperatures (Lillehaug et al. 2003; Samuelsen et al. 2006). These are expected to become more abundant with increased temperature and occur more frequently throughout the year. However, diseases such as winter ulcers and cold-water vibriosis are associated with low temperatures and should become less frequent with higher temperature. In addition, some parts of the immune system may function more effectively at higher temperatures and better resist infections (Le Morvan et al. 1996; Eggset et al. 1997). Most disease outbreaks occur at extreme temperature events. The increased incidence of periods with high temperature will increase the risk of disease (Bergh et al. 2007). Climate change may also shift the distribution of particular pathogens adapted to specific temperature ranges. Some exotic diseases may appear and others disappear.

The occurrence and growth of parasites common in aquaculture also depend on temperature. Higher temperatures lead to a shorter generation time, higher production of parasites, and subsequent production losses and increasing mitigation costs. However, many parasites have complex life cycles, hence making it difficult to predict the actual effect from increased temperature. Different species also have different temperature ranges that they thrive within. Increasing temperature could hence result in redistributions of parasite populations. Sea lice (*Lepeophtheirus salmonis*), the most common salmon parasite (Boxaspen 1997), is currently more problematic in the southern, warmer areas than in the Arctic. Sea lice spread depends on current patterns and larval stage. These are influenced by increased temperature and expected increase in freshwater runoff. The combined effects of sea lice are difficult to predict. However, infections will probably increase, resulting in higher costs for treatment, reduced productivity of farmed fish and higher infection rates among wild salmon (Bergh et al. 2007).

#### Algal blooms and precipitation

Increased precipitations will probably lower coastal water salinity, strengthen the stratification and influence nutrient concentrations. Changing zooplankton communities that graze on phytoplankton further increase system complexity and make predictions difficult (Gubbins 2006). Changes in temperature may shift algal community towards flagellates and dinoflagellates, some of which could harm farmed fish and shellfish (Sætre et al. 2003). Other algal groups could also grow but the resulting algal community and their dynamics are difficult to foresee.

Increased precipitation in Norway (Bergh et al. 2007) and the resulting increased river discharges could strengthen stratification in the fjords. The stronger freshwater stratification will result in higher temperatures in the fjords and the increased runoff will increase surface currents. Increased land runoffs will trigger higher nutrient discharges. However, direct impacts from changes in precipitation are likely to be small: smolt production may benefit from increased rainfall, which improves freshwater supply from rivers in May/June. The indirect effect on water temperature will exacerbate the effects described earlier. Overall the farms should directly benefit from the provision of oxygenated water and waste product removal from the cages.

#### Ocean acidification

The rise of atmospheric carbon dioxide content is expected to lower oceanic pH by 0.3 to 0.5 and carbonate saturation by about 45% (IPCC 2007; Andersson et al. 2008). Fish are well adapted to changes in ocean acidity, so direct impacts will be small for this species group, while indirect ecosystem impacts could affect fish food (see below, Callaway et al. 2012). Lower pH will mainly impact on organisms with calcium shells or skeleton, hence all farmed shellfish species particularly during their early life stages (Allison et al. 2011; Callaway et al. 2012). Shellfish culture is a marginal share of current Arctic aquaculture so the socioeconomic impacts should be small, except for future potential expansion of shellfish farming—in the Arctic and elsewhere (Allison et al. 2011).

#### Feed resources

Aquaculture is increasingly connected to global resource systems through feed resources: smaller pelagic species are major ingredients to produce fishmeal and fish oil for fish feed (Klinger and Naylor 2012; Cao et al. 2015). Arctic fishing fleets increasingly target Arctic pelagic species for human consumption instead of fish meal and oil production, for example, the Norwegian spring spawning herring, the capelin in the Barents Sea and in Icelandic and Greenland waters. Climate change could indirectly influence aquaculture if it affects important inputs for aquafeeds like the Peruvian anchoveta (*Engraulis ringens*) that dominates global fishmeal and fish oil production. Most aquaculture species (fish and crustaceans) increasingly feed on terrestrial crops instead of feed from the sea (Troell et al. 2014), which further increases the vulnerability to climatic effects on land far away from the actual farming areas.

#### Management, opportunities and adaptation

In the Arctic, the minimum water temperature for economically sustainable farming may limit the available area for farming. Temperature increase will increase this area for species not reaching the upper temperature bound. Higher water temperature would make more ice-free sites available, a necessity for cage farming. With increased water temperature, new species with higher temperature optima could be introduced. Species farmed in sub-Arctic areas could indicate possible future Arctic farming species and volumes. Along the southern coast of Alaska, shellfish and aquatic plants dominate farming but with limited production (sales value of about 400 000 USD in 2010). Small farming volumes could expand into the current Arctic. However, Alaska has banned finfish farming. Lifting this ban could trigger the introduction of fish farms in the current farming areas and into the Arctic.

In Canada, Atlantic salmon is the main species at USD 192 million in 2014 (FAO 2016), but some shellfish culture occurs in the northern parts of Quebec and Newfoundland. The industry will likely expand northwards. However, reaching as far as the current Arctic requires relatively large increases in temperature.

Iceland has no close “neighbours” that can inform future potential aquaculture species expansion. The Norwegian and Russian Arctic are likely to follow the current activities in the remaining part of Norway, focusing mainly on salmon. Norway hosts a considerably higher production of rainbow trout in the south and more Atlantic halibut (Table 3). Both these species have a higher temperature preference than salmon and are likely to be farmed in the Arctic in the future. Oysters like the European oyster (*Ostrea edulis*) and scallops (*Pecten maximus*) are primarily grown in warmer waters and the anticipated warming may not be sufficient to bring temperatures in the Arctic to comparable levels.

#### Economic implications

Socioeconomic impacts from climate change could be significant for the aquaculture sector. However, these effects are also linked with changes in other economic sectors in the Arctic, and in the rest of the world so it is difficult to foresee how the overall effects will play out (Crépin et al. 2017). Most economic models have focused on the impacts on aquaculture related to increased temperature. Some advanced models target salmon aquaculture in Norway (Lorentzen and Hanneson 2005, 2006; Lorentzen 2008 and Steinshamn 2009). Alternative development scenarios for Norwegian salmonid culture with and without warming highlight a positive effect on fish growth from increased water temperature. Sales prices could, however, decrease due to an expected increase in overall Norwegian salmon production (Lorentzen and Hanneson 2005). Increased temperature resulted, in all modelled cases, in higher slaughter weight and more frequent optimal harvest timing (Lorentzen and Hannesson 2006; Steinshamn 2009). Legal requirement currently limits potential production sites in Norway and thus forces adaptation on site to climate change effects. A license is typically granted for one region and cannot be transferred to any of the other four regions in place for aquaculture management. Model predictions also show a significant improvement in productivity for the northern farms and vice versa for the farms furthest south and a corresponding northward shift in production if the restrictions are lifted (Hermansen and Heen 2012).

## Conclusions: an outlook for future arctic seafood production

Current Arctic fisheries mainly operate in the Northeast Atlantic, with some fishing, whaling and sealing activities in the Northwest Atlantic. This review indicates that substantial spatial and temporal variability already characterise these fisheries and climate change will likely exacerbate these. According to IPPC, “*Nations at higher latitudes may benefit from climate change effects on ocean ecosystems, at least initially*” (IPCC 2014). Also, “*Increased variability could increase tensions among fishing nations creating climate change*-*related conflicts like the recent conflict over Atlantic mackerel stocks, previously shared between EU and Norway but now also targeted by Icelandic fishermen* – *a response to mackerel stocks migrating into the Icelandic economic exclusive zone during summertime*” (IPCC 2014). The management regimes for fisheries seem relatively robust, despite some tensions in the wake of re-distributed fish stocks. These tensions can, however, put great pressure on existing regulatory regimes, especially when quota distribution depends on historical rights and the fish distribution turns out differently in the fishing right zones of the coastal nations. Norway is by far the dominant aquaculture producer in the Arctic. Salmon production in this region provides important employment opportunities even though the emergent farming structure relies less on local employments.

The climate-induced temperature rise on the Norwegian coasts is likely to range between 0.5° and 2.5° and play out differently during different seasons. Despite large uncertainties, and just a few detailed studies that specifically target climate change impacts on Arctic aquaculture, the direct effects of a temperature change on the aquaculture industry can be modelled with fairly good accuracy, including effects on fish growth and impacts on the whole industry. These models indicate positive effects from warming water temperatures on Arctic aquaculture. Direct effects related to storm frequencies and intensities can be relatively well anticipated, but with high uncertainty. Other indirect effects, such as diseases and pest species and freshwater runoff, are much harder to predict. However, it is certain that the environmental conditions will change and that the industry will have to adapt to these changes. For enabling the industry to do so, there is a need to look over existing regulatory frameworks and start a multi-stakeholder dialogue to find out where and how aquaculture operations can move or change their operations. As the Arctic Region is undergoing multiple changes, involving changes in economic conditions and large-scale environmental changes, the different ways that aquaculture in the Arctic can adapt will be linked to the overall changes occurring in the region. Thus, a broader integrative approach is needed for successful governance of the Arctic system (Crépin et al. 2017).

## Footnotes

1 Based on statistics from Statistics Norway (http://www.ssb.no) and Norwegian Directorate of Fisheries (http://fiskeridir.no).

2 Based on fishery statistics from Statistics Iceland (http://www.hagstofa.is/).

3 Iceland Statistical yearbook for 2013 (http://www.stat.gl/publ/da/SA/201307/pdf/2013%20statistisk%20%C3%A5rbog.pdf).

4 Anon (2013), Boboedova (2014), Moran (2013) and Stammler-Gossmann (2014) report figures in a similar order of magnitude for 2011, 2012, and 2013.

5 Russian coastal vessels tend to have larger crews and stay at sea much longer than for example Norwegian “coastal” fishing vessels. (Vilhjálmsson and Hoel, 2004, p. 702).

6 Russian official catch statistics (http://fish.gov.ru/activities/Documents/f407-0.pdf, in Russian) report 1 million tonnes of total 4 million tonnes caught in the Northeast Atlantic in 2013.

7 Moran (2014) reports instead 12.5% (520 000 tonnes) of total 4.15 million tonnes Russian landings caught in the Northeast Atlantic 2013. MRG (2015) reports landings in the Murmansk region of 700 000 tonnes in 2013, an increase of 23 per cent from 2012 (571 000 tonnes). Catch statistics from ICES show that Russian catch amounted to 950 000 tonnes in 2012, of which 807 000 tonnes were caught in the Arctic parts (area I, II, Va or XIVa).

8
www.gks.ru/bgd/regl/b14_12/IssWWW.exe/Stg/d01/16-01.htm.

9 See Statistics Greenland (http://www.stat.gl/publ/da/NR/201402/pdf/Produktionsbaseretnationalregnskab2003-2013.pdf).
